# Supplementary material for: The Dialogic Health Systems Research Framework (DHSRF): A tool for facilitating self-criticality, researcher interactions and knowledge management in Health Systems Research & Policy Studies
Source: PLOS Glob Public Health. 2025 Sep 19;5(9):e0004209. doi: 10.1371/journal.pgph.0004209 (PMC12449027; doi:10.1371/journal.pgph.0004209)
Supplement: S2 File — (PDF) [file pgph.0004209.s002.pdf]

Supplementary 2: Definition of HSR

The working paper for WHO Global Strategy on Health Systems Research by Hoffman et al [1] defines HSR as "a multidisciplinary field of health research which studies governance, financial and delivery arrangements for health care and public health services, implementation considerations for reforming or strengthening these arrangements, and broader economic, legal, political and social contexts in which these arrangements are negotiated and operate. Health systems research aims to improve the understanding and performance of health systems. Health systems research includes all of health services research, most health policy research, and some clinical and population health research, but does not include any biomedical research." It sets out the need to simultaneously address micro, meso and macro level dimensions.

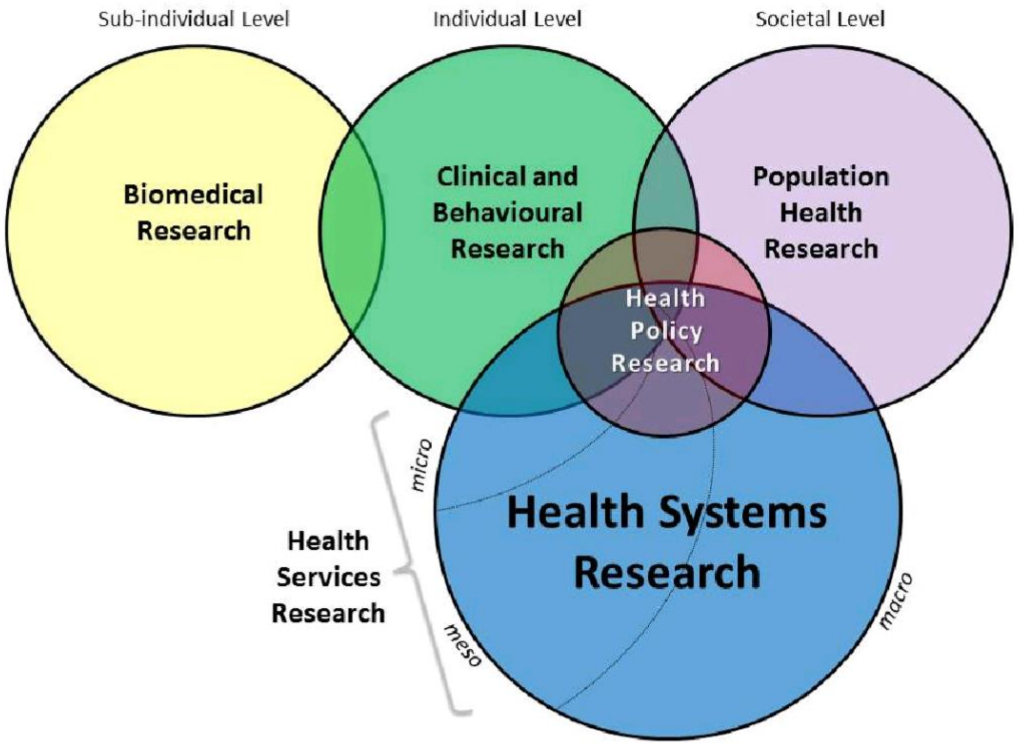

Figure 1. Health Systems Research as a Multidisciplinary Field of Health Research (Source: Hoffman et al., 2012)

The Alliance for Health Policy and Systems Research (AHPSR), gaining prominence in the 2000s, define their proposed iteration of HSR, Health Policy and Systems Research (the nomenclature of the field adopted also by WHO as a member of this alliance), as a field " … that seeks to understand and improve how societies organize themselves in achieving collective health goals, and how different actors interact in the policy and implementation processes to contribute to policy outcomes. By nature, it is interdisciplinary, a blend of economics, sociology, anthropology, political science, public health and epidemiology that together draw a comprehensive picture of how health systems respond and adapt to health policies, and how

health policies can shape − and be shaped by − health systems and the broader determinants of health." [2]

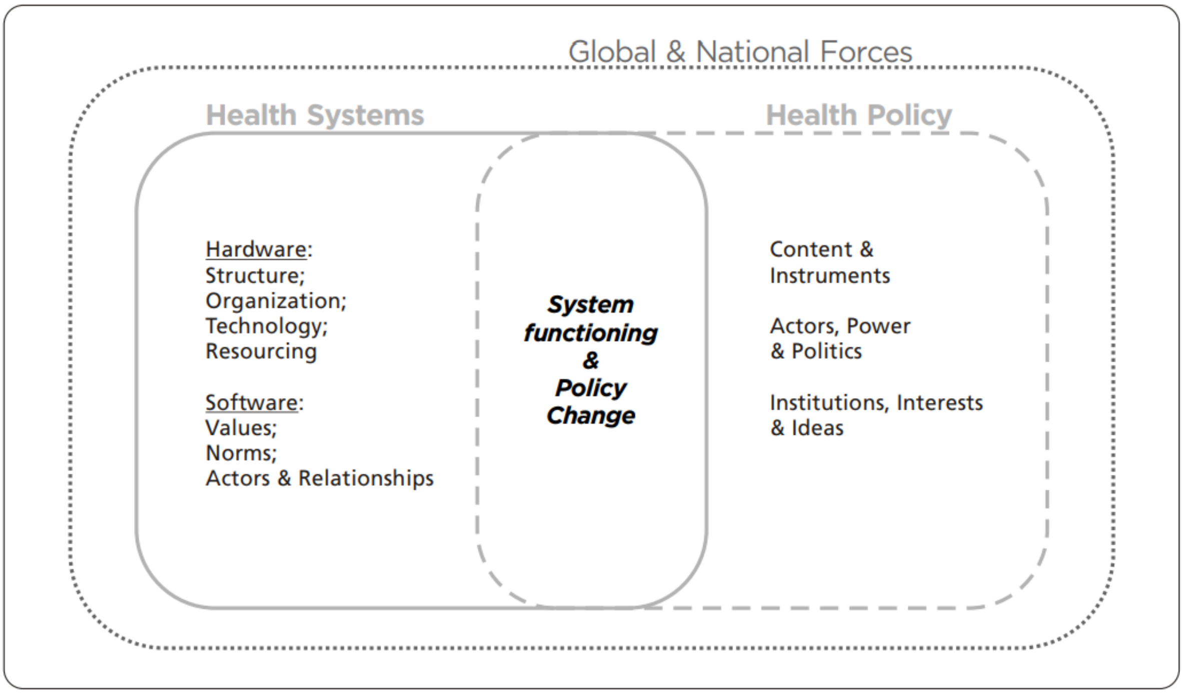

Figure 2. The terrain of Health Policy and Systems Research (Source: Gilson, 2012)

While we broadly agree with Hoffman et al's definition, including that bio-medical research is outside the remit of HSR and Policy Studies, we believe that examining 'the *system* of biomedical research' needs to be explicitly included within HSR. Epidemiology, the cornerstone of public health, includes biomedical research besides other dimensions. Both biomedical and clinical research directly influence people's health through health technologies and other interventions that are delivered through health systems. The biomedical research system thus should be considered an important component of HSR.

We also agree with Hoffman et al that health policy research is part of but also partially outside HSR, differing from the Alliance's definition of HPSR, which puts Health Policy as the central objective of HSR. In our view, HSR and PS are two closely related and often overlapping, yet distinct interdisciplinary fields with widely differing methodological imperatives. Health Policy is a crucial part of HSR, but that is not the only imperative of HSR. Policy studies (PS) is one of the many components of HSR, with implementation research, evaluation research, and the socio-cultural dimensions of health care as other major components. Epidemiology, HSR, PS, and the exploration of the dynamic SPEC (socio-political-economic-cultural) contexts, represent the essential pillars of public health research. The field of HSR draws from all these, depending on the purpose of the research and research questions. HSR is essentially meant to give a deeper understanding of health systems and their dynamics, which also generates the evidence for policy making, planning and implementation. Policy studies (a field that attempts to analyse the content of health policies and understand the process of policy

formulation) needs to be distinguished from the actual process of policy formulation using HSR evidence as two separate activities, each with their own relevance and imperatives.

While HSR is mainly concerned with the technical and operational dimensions of planning and implementation including those components that are outside the state's purview and practiced as 'informal systems', Health Policy Studies are more oriented towards the political dimensions and processes of public policy making concerning improvement of population health and the various systems/sub-systems that may be involved. Therefore, we prefer to keep them linked and yet stand apart as HSR & PS.

While HPSR focuses on the institutional policy aspect of HSR, other branches may give primacy to people's health-seeking behaviour and health care practices in specific settings. We, for instance, adopt a socio-cultural approach to define health systems as constitutive of the systemic determinants of health in a population that generate its health and morbidity profile as well as the health care systems (formal and informal) developed to maintain health and deal with ill health. The socio-cultural dimensions form the context in which the institutional systems perform, and which they influence, the formal itself being a socio-cultural entity. This socio-cultural dimension of the health system tends to be the least examined in HSR despite it being recognised as a significant barrier and facilitator of policy formulation and implementation. Here we use HSR as a composite of all of these components, and when we need to focus on the policy aspects separately, we prefer to make the distinction by expanding it to HSR & PS.

Our understanding of the composite/comprehensive HSR differs from the techno-managerial approach and adopts a socio-cultural approach that also includes the techno-managerial dimensions in its ambit. It is as follows:

A. Health System Approaches

- **Techno-managerial approach:** An approach to health systems that limits its focus to the organisation of technology-based health care and management of formal delivery systems, with minimal attention to social dimensions and processes shaping health and health care. [3]
- **Comprehensive/Socio-cultural approach:** An approach to health systems that addresses the different societal arrangements that exist for maintaining and improving people's health, including the formal and informal arrangements. These include macro, meso and micro level arrangements related to food, hygiene, sanitation, physical exercise, leisure and social relationships, expression of emotions, etc., in addition to the specific practices for prevention of disease and for promoting health, treating ill-health and easing physical and mental suffering, thereby going well beyond consideration of the formally organised health services. It includes the study of the formal health services as social institutions embedded in this socio-cultural context. [4]

B. Health system definition:

- **Techno-managerial definition:** A health system consists of all organisations, people and actions whose primary intent is to promote, restore or maintain health. [5]
- **Socio-cultural definition:** The systemic determinants of health in a population that generate its health and morbidity profile as well as the health care systems developed to maintain health and deal with ill health.

C. Adopting the Socio-cultural definition, health systems constituents are as follows:

- **Health care system:** The health care system is a whole of political, economic and cultural, technical and organizational factors, relations, processes and elements, in which individuals, groups and communities interrelate, having the goal to satisfy their health needs. [6] It includes the two components defined below, but also the wider dimensions that societies address to improve their health and wellbeing.
- **Health service system:** Health services are any service (i.e. not limited to medical or clinical services and including both formal and informal services) aimed at contributing to improved health or the diagnosis, treatment and rehabilitation of sick people. [7] It includes the component given below and also other health related interventions such as public health measures and services of other/traditional health knowledge systems.
- **Medical care system:** The component of the health service system that is organised around the delivery of medical services.

References

[1] Hoffman, S. J., Røttingen, J. A., Bennett, S., Lavis, J. N., Edge, J. S., & Frenk, J. Background paper on conceptual issues related to health systems research to inform a WHO global strategy on health systems research. Health Systems Alliance [Internet]. 2012; Available from:
https://www.academia.edu/65618679/Background_Paper_on_Conceptual_Issues_Related_to_Health_Systems_Research_to_Inform_a_WHO_Global_Strategy_on_Health_Systems_Research

[2] Gilson, L., & World Health Organization. Health policy and system research: a methodology reader: the abridged version [Internet]. Genève, Switzerland: World Health Organization; 2013. Available from: https://iris.who.int/handle/10665/44803

[3] Luning, P.A. & Marcelis, W.J. A food management research methodology integrating technological and managerial theories. Trends in Food Science & Technology [Internet]. 2009;20(1):35–44. Available from: https://doi.org/10.1016/j.tifs.2008.09.013

[4] Purola T. A Systems Approach to Health and Health Policy. Medical Care. 1972;10(5):373–9. Available from: http://www.jstor.org/stable/3763004

[5] World Health Organization. Monitoring the Building Blocks of Health Systems: A Handbook of Indicators and their Measurement Strategies. Genève, Switzerland: World Health

Organization; 2010. Available from: https://apps.who.int/iris/bitstream/handle/10665/258734/9789241564052-eng.pdf

[6] Health Care as a System: Elements, Boundaries, Levels. In: Management in Health Care Practice: A Handbook for Teachers, Researchers and Health Professionals [Internet]. Zagreb: Hans Jacob Publishing Company; 2008. Available from: https://www.biejournals.de/index.php/seejph/article/download/1910/pdf_4/6732

[7] World Health Organization. Health Systems Strengthening Glossary [Internet]. World Health Organization. Available from: https://www.who.int/docs/default-source/documents/health-systems-strengthening-glossary.pdf
