# Supplementary material for: The Dialogic Health Systems Research Framework (DHSRF): A tool for facilitating self-criticality, researcher interactions and knowledge management in Health Systems Research & Policy Studies
Source: PLOS Glob Public Health. 2025 Sep 19;5(9):e0004209. doi: 10.1371/journal.pgph.0004209 (PMC12449027; doi:10.1371/journal.pgph.0004209)
Supplement: S3 File — (PDF) [file pgph.0004209.s003.pdf]

Supplementary 3. Glossary

The glossary contains standard definitions of most terms as found in the literature. In some cases they have been modified by the research team based on review and analysis to appropriately reflect their positions. Those using the DHSRF can use these definitions to create their own operational definitions as relevant to their research.

1. **Health Systems Research Concepts**

   A. **Health System Approaches**

   - **Techno-managerial approach:** An approach to health systems that limits its focus to the organisation of technology-based health care and management of formal delivery systems, with minimal attention to social dimensions and processes shaping health and health care.[1]
   - **Comprehensive/Socio-cultural approach:** An approach to health systems that addresses the different societal arrangements that exist for maintaining and improving people's health, including the formal and informal arrangements. These include macro, meso and micro level arrangements related to food, hygiene, sanitation, physical exercise, leisure and social relationships, expression of emotions, etc., in addition to the specific practices for prevention of disease and for promoting health, treating ill-health and easing physical and mental suffering, thereby going well beyond consideration of the formally organised health services. It includes the study of the formal health services as social institutions embedded in this socio-cultural context.[2]

   B. **Health system definition:**

   - **Techno-managerial definition:** A health system consists of all organisations, people and actions whose primary intent is to promote, restore or maintain health.[3]

---

[1] Luning, P.A. & Marcelis, W.J. (2009). A food management research methodology integrating technological and managerial theories. Trends in Food Science & Technology, 20(1), 35-44. https://doi.org/10.1016/j.tifs.2008.09.013

[2] Purola, T. (1972). A Systems Approach to Health and Health Policy. Medical Care, 10(5), 373–379. http://www.jstor.org/stable/3763004

[3] World Health Organization. Monitoring the Building Blocks of Health Systems: A Handbook of Indicators and their Measurement Strategies. Genève, Switzerland: World Health Organization; 2010. Available from: https://apps.who.int/iris/bitstream/handle/10665/258734/9789241564052-eng.pdf

- **Socio-cultural definition:** The systemic determinants of health in a population that generate its health and morbidity profile as well as the health care systems developed to maintain health and deal with ill health.

Adopting the Socio-cultural definition:

- **Health care system:** The health care system is a whole of political, economic and cultural, technical and organizational factors, relations, processes and elements, in which individuals, groups and communities interrelate, having the goal to satisfy their health needs.[4]
- **Health service system:** Health services are any service (i.e. not limited to medical or clinical services and including both formal and informal services) aimed at contributing to improved health or the diagnosis, treatment and rehabilitation of sick people.[5]
- **Medical care system:** The component of the health service system that is organised around the delivery of medical services.

C. **Health System Frameworks** - A bird's eye view of the health system that defines, describes and explains the health system, its objectives, structural and organizational elements, functions and processes.[6]

Health System Research Framework - A framework that elucidates the major components of health systems research (research objective, health systems conceptualisation, methodological approach, data analysis and findings,

---

[4] Kovačić, L. & Jakšić, Z. "Health Care as a System: Elements, Boundaries, Levels", in Kovačić, L & Zaletel-Kragelj, L. (Ed.) Management in Health Care Practice: A Handbook for Teachers, Researchers and Health Professionals, 2008, Hans Jacob Publishing Company: Zagreb, https://www.biejournals.de/index.php/seejph/article/download/1910/pdf_4/6732

[5] World Health Organisation, Health Systems Strengthening Glossary, https://www.who.int/docs/default-source/documents/health-systems-strengthening-glossary.pdf

[6] Shakarishvili, G., Atun, R., Berman, P., et.al Converging Health Systems Frameworks: Towards A Concepts-to-Actions Roadmap for Health Systems Strengthening in Low and Middle Income Countries. Global Health Governance, 2010, Vol. III, No. 2, http://blogs.shu.edu/wp-content/blogs.dir/109/files/2011/11/Shakarishvili-et-al_Converging-Health-Systems-Frameworks_Spring-2010.pdf

recommendations, and values and principles underlying all these) and examines their interlinkages and relationships and assesses their coherence across the research.

D. **Types of Health Systems Research**

- **Evaluation research -** A research project that has as its focus the evaluation of some program process, policy or product. Unlike program evaluation, evaluation research is intended to generate knowledge that can inform both decision-making in other settings and future research.[7]
- **Implementation Research –** Implementation research is a type of health policy and systems research concerned with the study of clinical and public health policies, programmes, and practices, with the basic intent being to understand not only what is and isn't working, but how and why implementation is going right or wrong, and to test approaches to improve implementation.[8]
- **Policy Analysis** – Policy Analysis is the process of identifying potential policy options that could address your problem and then comparing those options to choose the most effective, efficient, and feasible one.[9]
- **Policy Studies-** It is an inquiry into the system of generating policies/a specific policy, its content, determinants, actors and processes. It also includes the outcomes of the policy, if implemented.[10]
- **Research/Scientific Synthesis** - Research or scientific synthesis is the integration and assessment of knowledge and research findings pertinent to a particular issue with the aim of increasing the generality and applicability

[7] Canadian Institute of Health Research, A Guide to Evaluation in Health Research, https://cihr-irsc.gc.ca/e/documents/kt_lm_guide_evhr-en.pdf

[8] Peters D.H., Tran, N.T., Adam, T. Implementation Research in Health: A Practical Guide, Alliance for Health Policy and Systems Research, World Health Organization, 2013. https://iris.who.int/bitstream/handle/10665/91758/9789241506212_eng.pdf

[9] Centers for Disease Control and Prevention, https://www.cdc.gov/policy/polaris/policyprocess/policyanalysis/index.html

[10] M. Weible, C., & Sabatier, P.A. (Eds.). (2018). Theories of the Policy Process (4th ed.). Routledge. https://doi.org/10.4324/9780429494284

of, and access to, those findings.[11][12] The complexity of health systems requires that HSR is interdisciplinary and uses multiple sources and nature of evidence which needs to be synthesized for addressing HSR questions. Therefore, this requires going beyond aggregation of multiple study findings to synthesize multiple kinds of evidence into a logical and contextualised whole.[13]

E. **Nature of Disciplinary Interaction**[14]

- **Multidisciplinarity** - Multidisciplinarity draws on knowledge from different disciplines but stays within the boundaries of those fields.
- **Interdisciplinarity** - Interdisciplinarity analyzes, synthesizes and harmonizes links between disciplines into a coordinated and coherent whole.
- **Transdisciplinarity** - Transdisciplinarity integrates disciplinary and lay insights towards co-production of knowledge and in so doing transcends traditional disciplinary boundaries.

F. **Research Coherence:** Research Coherence “describes the fit between the aim, the philosophical perspective adopted, and the researcher role in the study as well as the methods of investigation, analysis and evaluation undertaken by the researcher.”[15]

G. **Health System Research Paradigms**

[11] Hampton, S.E. & Parker, J.N. (2011) Collaboration and Productivity in Scientific Synthesis, BioScience, 61(11), 900–910, https://doi.org/10.1525/bio.2011.61.11.9; Magliocca, N. R., et.al (2015). Synthesis in land change science: methodological patterns, challenges, and guidelines. Regional environmental change, 15, 211-226;

[12] Baron, J.S., et.al (2017). Synthesis Centers as Critical Research Infrastructure, BioScience, 67(8), 750–759, https://doi.org/10.1093/biosci/bix053

[13] Langlois, E.V., Daniels, K., Akl, E.A, editors. Evidence synthesis for health policy and systems: a methods guide. Geneva: World Health Organization; 2018. https://apps.who.int/iris/bitstream/handle/10665/275367/9789241514552-eng.pdf

[14] Choi, B.C.K & Pak, A.W.P. (2006). Multidisciplinarity, interdisciplinarity and transdisciplinarity in health research, services, education and policy: 1. Definitions, objectives, and evidence of effectiveness. Clinical and investigative medicine, 29(6), 351-64. https://pubmed.ncbi.nlm.nih.gov/17330451/

[15] Vaismoradi, M., & Salsal, D. mahvash. (2011). Coherence in qualitative research. *Advances in Nursing & Midwifery*, *20*(70), 45–53. Retrieved from https://journals.sbmu.ac.ir/en-jnm/article/view/2047

- **Paradigm -** A paradigm constitutes a set of theories, assumptions, and ideas that contribute to one's worldview and approach to engaging with other people or things. It is the lens through which a researcher views the world and examines the methodological components of their research to make a decision on the methods to use for data collection and analysis.[16]
- **Research paradigms -** Practical application of the various elements of the paradigm in health systems research. Research paradigms consist of four philosophical elements: axiology, ontology, epistemology, and methodology.[17]
    - Axiology refers to the study of values and their role in shaping research (Values and principles undergirding HSR). It addresses the ethical and value-based considerations that influence a researcher's choices, including what is deemed important to study, the purpose of research, and how findings should be used.[18]
    - Ontology is the study of the nature of reality (nature of health systems in HSR), addressing questions about what exists and the nature of being. It concerns the assumptions a paradigm makes about the structure of reality.[19]
    - Epistemology defines the methods of inquiry, focusing on how knowledge is acquired, what counts as valid knowledge, and the relationship between the knower and the known (In HSR, for instance, Positivism emphasizes objective, empirical observation and testing, while interpretivism values subjective understanding through qualitative insights. Critical Holist, on the other hand, promotes knowledge pluralism and dialogic interaction). [20]

---

[16] Kivunja, C. & Kuyini, A.B. (2017) Understanding and applying research paradigms in educational contexts. International Journal of Higher Education. 2017;6(5):26-41. As cited in: Alele, F., & Malau-Aduli, B. (2023). An introduction to research methods for undergraduate health profession students. James Cook University. https://jcu.pressbooks.pub/intro-res-methods-health

[17] Creswell, J.W. Educational Research: Planning, Conducting, and Evaluating. 4th ed. W. Ross MacDonald School Resource Services Library; 2013

[18] Guba, E. G., & Lincoln, Y. S. (1994). Competing paradigms in qualitative research. In N. K. Denzin & Y. S. Lincoln (Eds.), Handbook of qualitative research (pp. 105–117). Sage Publications, Inc.

[19] Guba, E. G., & Lincoln, Y. S. (1994). Competing paradigms in qualitative research. In N. K. Denzin & Y. S. Lincoln (Eds.), Handbook of qualitative research (pp. 105–117). Sage Publications, Inc.

[20] Guba, E. G., & Lincoln, Y. S. (1994). Competing paradigms in qualitative research. In N. K. Denzin & Y. S. Lincoln (Eds.), Handbook of qualitative research (pp. 105–117). Sage Publications, Inc.

- Methodology refers to the systematic approaches and strategies used to collect, analyze, and interpret data. It is the practical application of ontological and epistemological assumptions informed by axiological moorings.[21]

These four elements inform the design and conduct of research projects (Figure 1.1), and a researcher would have to consider the paradigms within which they would situate their work before designing the research.[22]

- **Conventional public health/Pragmatist -** A paradigm relying on framing the health problem from an epidemiological and problem-solving approach, designing levels of prevention to deal with it from structural to the individual level. Linkages with societal processes and context are minimally defined with little social theory being employed. Primarily quantitative methods combined with tacit social and organisational knowledge. Epistemologically, this paradigm follows a pragmatist stance that is focused on practical application of knowledge to real-world settings.[23]
- **Positivist -** Positivism relies on the hypothetico-deductive method to verify a priori hypotheses that are often stated quantitatively, where functional relationships can be derived between causal and explanatory factors (independent variables) and outcomes (dependent variables).[24]
- **Realist -** This theoretical paradigm is informed by a branch of philosophy called Critical Realism that distinguishes between the 'real' world and the 'observable' world, giving importance to **context** which triggers certain **mechanisms** in turn producing observable **outcomes**. Using plural methodology and an explanatory approach (what works? for whom? and why? Similarly, for what does not work as

---

[21] Guba, E. G., & Lincoln, Y. S. (1994). Competing paradigms in qualitative research. In N. K. Denzin & Y. S. Lincoln (Eds.), Handbook of qualitative research (pp. 105–117). Sage Publications, Inc.

[22] Alele, F., & Malau-Aduli, B. . An introduction to research methods for undergraduate health profession students. James Cook University. 2023. https://jcu.pressbooks.pub/intro-res-methods-health)https://jcu.pressbooks.pub/intro-res-methods-health

[23] Creswell, J.W. Educational Research: Planning, Conducting, and Evaluating. 4th ed. W. Ross MacDonald School Resource Services Library; 2013

[24] Park, Y.S., Konge, L., Artino Jr, A.R. (2020). The Positivism Paradigm of Research. Academic Medicine 95(5), 690-694. https://journals.lww.com/academicmedicine/fulltext/2020/05000/the_positivism_paradigm_of_research.16.aspx

well) it explores mechanisms linked to actors and contexts mediating cause and effect.[25]

- **Realist closer to Positivist** - Narrow boundary, context-mechanism-outcome with minimal attention to context and linkages; mixed methods with greater role of the quantitative; Top-down.
- **Realist closer to Holist** - Wider boundary conceptualised for the context-mechanism-outcome configurations; Uses mixed methods with greater role of the qualitative. Considers context and linkages with a minimum of one and maximum of three of the following: Historical analysis, Epidemiological orientation, Knowledge pluralism and Bottom-up or combined system vantage point.
- **Critical Holist** - 'Holism' is a term much used in relation to health. In relation to health knowledge, at the individual level, it means that the mind and the body are intrinsically intertwined, and the individuals are embedded in their ecological and social context. At the societal level, holism means that communities are not simply aggregates of individuals but are greater than the sum of the individuals, societies are not just aggregates of communities but greater than their sum due to their inter-relationships. Similarly, organizations are not an aggregate of their structure alone but of their value frames, the interactive processes among those who comprise them and their interactions with their social context.[26] Critical theory, on the other hand, refers to empirically informed analysis of society's diversity, conflicts and contradictions for a deeper understanding of social processes and possibilities of just transformation.[27]

  Critical holism is thus an analytical approach that examines systems as interconnected wholes while evaluating the varied components of the system and their relationships. This perspective, in the vein of critical theory, highlights the diversity of social perspectives and foregrounds the vantage point of the

---

[25] University of Warwick, https://warwick.ac.uk/fac/soc/ces/research/current/socialtheory/maps/criticalrealism/#:~:text=Critical%20Realism%20(CR)%20is%20a,perceptions%2C%20theories%2C%20and%20constructions

[26] Ritu Priya, 2021. Critical Holism as Public Health Theory: Towards a Unifying Framework for Research, Policy and Planning. Dialogue: Science, Scientists and Society, Vol. 4, Special Issue on Public Health. DOI: https://doi.org/10.29195/DSSS.03.01.0033

[27] Stanford Encyclopedia of Philosophy. Critical Theory (Frankfurt School). https://plato.stanford.edu/entries/critical-theory/

marginalised by integrating critical thinking to question assumptions, power dynamics, and biases within the system.

As a research paradigm in HSR, Critical Holism makes use of these critical approaches while incorporating all the following — Historical analysis, Epidemiological orientation, Knowledge pluralism and Bottom-up or combined system vantage point, in addition to the Realist attributes.

2. Steps of the DHSRF

A. Values and Principles for Health Systems

- **Sustainability (Financial sustainability of program/economic viability, environmental integrity and social justice/equity/health outcome) -** The potential for sustaining beneficial outcomes for an agreed period at an acceptable level of resource commitment within acceptable organizational and community contingencies.[28] A sustainable health system improves population health by continually delivering the key functions of providing services, generating resources, financing and stewardship, incorporating principles of financial fairness, equity in access, responsiveness and efficiency of care, and does so in an environmentally sustainable manner.[29]

- **Equity -** (i) the absence of systematic or potentially remediable differences in health status, access to healthcare and health-enhancing environments, and treatment in one or more aspects of health across populations or population groups defined socially, economically, demographically or geographically within and across countries. (ii) a measure of the degree to which health policies are able to distribute well-being fairly.[30]

---

[28] WHO, Health Systems Strengthening Glossary, https://www.who.int/docs/default-source/documents/health-systems-strengthening-glossary.pdf

[29] Gocke, D., Johnston-Webber, C., McGuire, A., & Wharton, G. "Building Sustainable and Resilient Health Systems: Key Findings from Country Reports", Partnership for Health System Sustainability and Resilience (PHSSR). May 2023, https://www3.weforum.org/docs/WEF_PHSSR_Building_Sustainable_and_Resilient_Health_Systems_2023.pdf

[30] WHO, Health Systems Strengthening Glossary, https://www.who.int/docs/default-source/documents/health-systems-strengthening-glossary.pdf

- **Context-Appropriateness -** Context is conceptualized as a set of characteristics and circumstances that consist of active and unique factors that surround the implementation. As such it is not a backdrop for implementation but interacts, influences, modifies and facilitates or constrains the intervention and its implementation. Context is usually considered in relation to an intervention or object, with which it actively interacts. A boundary between the concepts of context and setting is discernible: setting refers to the physical, specific location in which the intervention is put into practice. Context is much more versatile, embracing not only the setting but also roles, interactions and relationships.[31]

- **People-centred care/People-centredness** - Care that is focused and organized around the health needs and expectations of people and communities rather than on diseases. People-centred care extends the concept of patient-centred care to individuals, families, communities and society. Whereas patient-centred care is commonly understood as focusing on the individual seeking care—the patient, people-centred care encompasses these clinical encounters and also includes attention to the health of people in their communities and their crucial role in shaping health policy and health services.[32]

- **Effectiveness -** Effectiveness is the extent to which a specific intervention, procedure, regimen or service, when deployed in the field in routine circumstances, does what it is intended to do for a specified population.[33]

- **Safety -** Within the broader health system context, it is a framework of organized activities that creates cultures, processes, procedures, behaviours, technologies and environments in health care that consistently and sustainably lower risks, reduce the occurrence of avoidable

---

[31] Pfadenhauer, L., Rohwer, A., Burns, J., Booth, A., Lysdahl, K.B., Hofmann, B., et al. (2016) Guidance for the assessment of context and implementation in health technology assessments (HTA) and systematic reviews of complex interventions: the Context and Implementation of Complex Interventions (CICI) framework. INTEGRATE-HTA Consortium, http://www .integrate-hta.eu/downloads/

[32] WHO, Health Systems Strengthening Glossary, https://www.who.int/docs/default-source/documents/health-systems-strengthening-glossary.pdf

[33] WHO, Health Systems Strengthening Glossary, https://www.who.int/docs/default-source/documents/health-systems-strengthening-glossary.pdf

harm, make error less likely and reduce impact of harm when it does occur.[34]

- **Clinical rationality -** Rationality is commonly defined as decision making that helps us achieve our goals. In the context of clinical medicine, this typically means the desire to improve our health. Rationality does not guarantee that a decision is error free; rather, rational decision-making accounts for the potential consequences of possible errors of action—false negatives and false positives—to help arrive at optimal outcomes.[35]

- **Ethical Practice -** This is concerned with the obligations and practices of the health care professionals and institutions to the patient and society.[36]

- **Appropriate Technology -** Appropriate technology is defined as the adaptation to local circumstances and conditions of knowledge and skills which are scientifically sound and acceptable to those who apply them and those for whom they are used. It should be affordable and should include appropriate use and effective interaction between service users and performers, as well as control of the cost and clinical benefits. Appropriate technology does not mean primitive or necessarily simple and/or less expensive. The initial cost should be considered within the context of the overall benefits and the expected outcome in the long run. Priority should be given to technologies improving public health services, with emphasis on equal access to health care for all.[37]

- **Technical Efficiency -** Technical efficiency is concerned with achieving maximum outputs with the least cost (monetary and time) and thereby informs health systems design.[38]

---

[34] World Health Organisation, https://www.who.int/news-room/fact-sheets/detail/patient-safety

[35] Djulbegovic, B., Elqayam, S. & Dale, W. (2018). Rational decision making in medicine: Implications for overuse and underuse. Journal of Evaluation in Clinical Practice, 24(3), 655-665. doi: 10.1111/jep.12851

[36] Markose, A., Krishnan, R. & Ramesh, M. (2016). Medical ethics. Journal of Pharmacy and Bioallied Sciences, 8(Suppl 1), S1-S4. doi: 10.4103/0975-7406.191934. https://www.ncbi.nlm.nih.gov/pmc/articles/PMC5074007/

[37] World Health Organisation, https://applications.emro.who.int/docs/em_rc44_tech_disc_1_en.pdf

[38] Akazili, J., Adjuik, M., Chatio, S., Kanyomse, E., Hodgson, A., Aikins, M. & Gyapong, J. (2008) What are the Technical and Allocative Efficiencies of Public Health Centres in Ghana? Ghana Medical Journal, 42(4):149-55. https://www.ncbi.nlm.nih.gov/pmc/articles/PMC2673839/

- **Affordability -** A system for financing health services so people do not suffer financial hardship when using them.[39] Health facilities, goods and services must be affordable for all. Payment for health-care services, as well as services related to the underlying determinants of health, has to be based on the principle of equity, ensuring that these services, whether privately or publicly provided, are affordable for all, including socially disadvantaged groups. Equity demands that poorer households should not be disproportionately burdened with health expenses as compared to richer households.[40]

- **Ecological Sensitivity -** Environmental sensitivity is related to an interest in the environment and presenting behaviours to protect it; it is a very important influence in ensuring sustainable development.[41]

- **Dignity in Care -** The right of individuals to be treated with respect as persons in their own right.[42]

- **Self-Reliance -** The capacity of individuals, communities or national authorities to take the initiative in assuming responsibility for their own health development and adopting adequate measures to maintain health that are understood by them and acceptable to them, knowing their own strengths and resources and how to use them and knowing when, and for what purpose, to turn to others for support and cooperation.[43]

- **Autonomy -** The right of patients to make decisions about their medical care without their health care provider trying to influence the decision. Patient autonomy does allow for health care providers to educate the patient but does not allow the health care provider to

---

[39] WHO, https://www.who.int/news-room/questions-and-answers/item/what-is-universal-health-coverage

[40] The measurement and monitoring of water supply, sanitation and hygiene (WASH) affordability: a missing element of monitoring of Sustainable Development Goal (SDG) Targets 6.1 and 6.2. New York: United Nations Children's Fund (UNICEF) and the World Health Organization, 2021, https://www.who.int/publications/i/item/9789240023284

[41] Yayla, O., Keskin, E. & Keles, H. (2022) "The Relationship Between Environmental Sensitivity, Ecological Attitude, and the Ecological Product purchasing Behaviour of Tourists" European Journal of Tourism, Hospitality and Recreation, 12(1), pp. 31-45. https://doi.org/10.2478/ejthr-2022-0002

[42] WHO Centre for Health Development (2004). A glossary of terms for community health care and services for older persons, https://apps.who.int/iris/handle/10665/68896

[43] WHO Centre for Health Development (2004). A glossary of terms for community health care and services for older persons, https://apps.who.int/iris/handle/10665/68896

make the decision for the patient.[44]

- **Empowerment –** In health promotion, empowerment is a process through which people gain greater control over decisions and actions affecting their health.[45] Community empowerment is a multilevel construct described for individuals, the organizations where they work, and their community settings. Individual empowerment, also referred to as psychological empowerment, refers to individuals gaining control over their lives with building up of self-confidence, boosting up of their self-esteem, critical awareness of their social context, and better decision-making capacity, leading to participation in change for improvement. Organizational empowerment deals with the potential ability of the organizations to influence societal change for improvement. Community empowerment deals with power relations and intervention strategies that enable communities to take control of the decisions that influence their lives and health.[46]

- **Trust -** Trust in health care is usually defined as a set of expectations that the patient has from the doctor and the healthcare system to help them heal. This set of expectations includes appropriate diagnosis, correct treatment, non-exploitation, genuine interest in the welfare of the patient and transparent disclosure of all information. Trust is like a forward-looking covenant between the doctor and the patient.[47]

- **Transparency -** The Institute of Medicine (IOM) defines healthcare transparency as making available to the public, in a reliable, and understandable manner, information on the health care system's quality, efficiency and consumer experience with care, which includes price and quality data, so as to influence the behavior of patients, providers, payers, and others to achieve better outcomes (quality and cost of care).[48]

---

[44] MedicineNet as cited in Harvard Health Publishing, https://www.health.harvard.edu/blog/take-control-of-your-health-care-exert-your-patient-autonomy-2018050713784

[45] WHO, Health Promotion Glossary of Terms 2021, https://www.who.int/publications/i/item/9789240038349

[46] Sai, T.S.R. & Prathap, S.S. Community Empowerment: Holistic Approach for Sustainable Improvements in Population Health. Indian Journal of Public Health, 2015:59(3):p 163-166. DOI: 10.4103/0019-557X.164647

[47] Gopichandran, V. & Chetlapalli, S.K. (2013). Dimensions and determinants of trust in health care in resource poor settings--a qualitative exploration. PLoS One, 8(7): e69170. https://www.ncbi.nlm.nih.gov/pmc/articles/PMC3712948/

[48] American College of Physicians. "Healthcare Transparency – Focus on Price and Clinical Performance Information", 2010. https://www.acponline.org/acp_policy/policies/healthcare_transparency_2010.pdf

- **Accountability** – The result of the process which ensures that health actors take responsibility for what they are obliged to do and are made answerable for their actions.[49]

- **Responsiveness -** Responsiveness entails reacting effectively to the needs and demands of the population and its different subpopulations and vulnerable groups. The content of the minimum package of activities should be informed both by the burden of disease and by the perceived needs of the population. It is a function of governance weighing the technical arguments; perceived needs; existing values and principles, and to decide which trade-offs to make, taking into account the infrastructure, level of development and capacity of implementation.[50]

- **Decentralization -** Political reform designed to promote local autonomy, decentralization entails changes in authority and financial responsibility for health services. Hence, decentralization can have a large impact on health service performance. There are several forms of decentralization affecting the health sector in different ways: (i) deconcentration, which transfers authority and responsibility from the central level of the Ministry of Health to its field offices; (ii) delegation, which transfers authority and responsibility from the central level of the Ministry of Health to organizations not directly under its control; (iii) devolution, which transfers authority and responsibility from the central level of the Ministry of Health to lower level autonomous units of government; (iv) privatization, which involves the transfer of ownership and government functions from public to private bodies, which may consist of voluntary organizations and for-profit and not-for-profit private organizations, with varying degree of government regulation.[51] Decentralization as an 'intervention' is often used in the health systems literature—as an arrangement in which

[49] UHC 2030, Health budget literacy, advocacy and accountability for universal health coverage Toolkit for capacity-building, https://www.uhc2030.org/fileadmin/uploads/uhc2030/2_What_we_do/2.3_Sharing_knowledge_and_networks/2.3.3_Civil_society_engagement/Health_Budget_Literacy/WHO013_UHC2030-capacity-building-toolkit_glossary.pdf
[50] World Health Organization (2000). The World Health Report 2000. Improving Performance, World Health Organization, Geneva. https://www.who.int/publications/i/item/924156198X
[51] World Health Organization, Health Systems Strengthening Glossary, https://www.who.int/docs/default-source/documents/health-systems-strengthenWHing-glossary.pdf

the power, resources or responsibilities are transferred from central to peripheral actors.[52]

B. Health System Conceptualisation

- **Boundary of the Health System:** The outer limits (context, institutions, capacities) within which the health system operates.[53]

- **Sub-Systems of the Health System**
  The subsystems constitute both formal and informal components of the health system. The structures and processes of these formal and informal health services represent their dynamic interactions, which, informed by their specific contexts, adapt and evolve through feedback loops, self-organization, emergent behaviour, and path-dependent trajectories, exhibiting both resilience and vulnerability to change. Adaptation and innovation in responding to social, economic, environmental, geographic and cultural shifts over time may also be observed.

- **Subsystems of the formal Health Service System[54] (Structures and Processes):** These include –
  - **Arrangements to address Ecological[55] and Social Determinants[56] -** The formal aspects of health services that involve structured policies and programs to tackle factors like environmental conditions, socioeconomic status, living and occupational conditions and access to resources that influence health outcomes. For instance, the health in all policies approach, Monitoring and regulation of environmental standards like access to clean

[52] Mills, A., Vaughan, J.P., Smith, D.L & Tabibzadeh, I. (1990). Health System Decentralization: Concepts, Issues and Country Experience. Geneva: World Health Organization
[53] World Health Organization, Health Systems Strengthening Glossary, https://www.who.int/docs/default-source/documents/health-systems-strengthening-glossary.pdf
[54] World Health Organisation, Health Systems Strengthening Glossary, https://www.who.int/docs/default-source/documents/health-systems-strengthening-glossary.pdf
[55] Watts, N., Amann, M., Arnell, et al. The 2020 report of The Lancet Countdown on health and climate change: responding to converging crises. Lancet (London, England), 2021;397(10269): 129–170. https://doi.org/10.1016/S0140-6736(20)32290-X
[56] Marmot, M. & R. Wilkinson (eds), Social Determinants of Health, 2nd edn (Oxford, 2005; online edn, Oxford Academic, 1 Sept. 2009), https://doi.org/10.1093/acprof:oso/9780198565895.001.0001

water, equity-focused models like mobile clinics bringing services to underserved areas, surveillance systems tracking social and ecological indicators to inform interventions etc.

- **Service delivery -** This encompasses the organisation, management and delivery of health care services ensuring equitable access, quality, safety, and continuity of care across various health conditions, levels of care and over the life-cycle.
- **Health Workforce** - This includes the training, deployment and support of different health care personnel—like administrative staff, nursing staff, doctors, paramedical staff, community health workers— engaged in different types of service provisioning.
- **Information/knowledge** - This focuses on the production, analysis, dissemination, and use of timely and reliable information to support decision-making and improve health outcomes.
- **Health technologies -** This involves ensuring equitable access to drugs, devices, medical and surgical procedures of assured quality, safety and efficacy.
- **Finance -** This is concerned with the mobilization, accumulation and allocation of money to fund the health systems, cover health needs of the people, protect people from health-related financial catastrophes in ways that improve quality, equity and efficiency.
- **Leadership & Governance** - This refers to the overall management and oversight of the health system including strategic policy development, coalition-building, regulation, attention to system design and accountability.
- **Community engagement** - This is the process of developing and maintaining relationships that enable stakeholders to work together, interfacing the community with the policymakers, implementers and health

care providers to achieve positive and sustainable health impact and outcomes.[57]

    - **Others** -Anything else that may be of interest in the given context of the study.

- **Informal Health Service System (Structures and Processes)** - Informal health services include health care that is provided outside of the regulated system, lacking formal recognition by the state. These include –
    - Informal providers of modern biomedicine
    - Traditional caregivers of codified traditional systems
    - Traditional caregivers of non-codified systems (Local Health Traditions)

- **Health system vantage point -** Analysis of the perspective underlying health systems research, planning and policymaking, which gives primacy to viewing the health system from either the bottom-up or top-down location. The vantage point influences which systemic values and dimensions are emphasized, how goals are set and what ways of achieving them are envisaged.
    - **Bottom-up vantage point** - A bottom-up or social/community vantage point encourages research, evaluation and planning beginning at the grassroots (community level) with primacy to the perceptions and practice of people who are meant to be the beneficiaries of the health system. It grants them rationality and agency and attempts to understand these and analyses the health system through these. For instance, in a study on preventive child health interventions, bringing forth the community's understanding of child health problems, their determinants and the perceived needs for improving child health together with their experience/perceptions of formal child health interventions would represent the bottom-up perspective and would inform the conceptualisation of the research problem and subsequent design of the study.

---

[57] World Health Organization. Community Engagement for Quality. https://qualityhealthservices.who.int/quality-toolkit/new-to-health-system-quality-thinking/community-engagement-for-quality

- **Top-down vantage point** - A top-down/institutional vantage point, on the other hand, gives primacy to formal institutions and activities and their functional attributes. It views the institutional values, structures and processes–from the perspective of planners, policy makers and implementers as the core of the health system and can go down to the community's experience of utilization of health services. From a top-down vantage point, for a study on preventive child health interventions, the prevailing policies and programmes and discrepancies in their implementation would inform the conceptualisation of the research problem.
- **A Combination** - Both Bottom-up and Top-down approaches are applied, contrasting or holistically bridging the two. Rationality of the institutional and the social is brought together in an interactive mode. The vantage point, in addition to community-focused studies would also apply to the nested subsystems of the health system as appropriate. For instance, a study on the challenges in the implementation of a TB program, conceptualising the problem based on the perceptions and perspectives of the peripheral health care providers like the DOTS workers and ASHA workers could represent the bottom-up perspective. The policymakers and planners, on the other hand, would represent the top-down perspective.

- **Dynamic Elements of a Health System and Its context**
  - **Ecosystem** - The dynamic complex of living organisms, their surroundings, and all their interrelationships contributing to people's health or the lack thereof.[58] For HSR, 'ecosystem' would refer to the natural and infrastructural environment in which people live and work or are exposed to, their interactions with these, and their influence on health status.
  - **Socio-political contexts of health and health care**- The combined social and political factors and their dynamics that influence people's health and well-being by shaping the conditions in which people live and work as well as their access to essential social and economic resources.

[58] Britannica, The Editors of Encyclopaedia. "ecosystem". Encyclopedia Britannica, 12 Feb. 2024, https://www.britannica.com/science/ecosystem. Accessed 26 February 2024

- **Meaning systems of health and health care** - The collective and individual health-related worldviews, experiences and perceptions about health, health problems and health care.
- **Health Culture** - Health culture covers a wide range of considerations which intimately interact with one another to form a sub-cultural complex. Perceptions of health problems, meanings ascribed to them and the response to these problems, in terms of knowledge, technologies and formation of various institutions to deal with various health problems and actual (health) behaviour of individuals and groups form this sub-cultural complex.[59]
- **Informal social arrangements & Community practices for health** - All informal societal arrangements for maximising health and minimising ill-health, shaped by agencies of different actors, their interactions and emergent behaviours. This includes informal care, people's health preserving and improving practices, and social support in ill-health or vulnerabilities. These would consist of health-seeking behaviour, ranging from self-care and household level care to resort to formal or informal health services; societal arrangements (e.g. for leisure, exercise, sports) and community practices (e.g. seasonal foods; maternal & childcare) that have a bearing on health, including emergent behaviour under specific contexts (e.g. household level health care practices during the COVID-19 pandemic).

- **Relationship of the elements across systems**- In complex systems, these dynamic elements are intersecting and interacting, thereby influencing health systems and their outcomes. These dynamics and their interrelationships need to be examined in HSR.
- **Theory of Change -** Theory of Change is "an outcomes-based approach which applies critical thinking to the design, implementation, and evaluation of initiatives and programs intended to support change in their context".[60] Critical thinking would involve examining

[59] Banerji, D. (1985). Health and Family Planning Services in India: An Epidemiological, Socio-cultural, and Political Analysis and a Perspective. Lok Paksh, New Delhi.
[60] Vogel I. Review of the Use of "Theory of Change" in International Development. London: Commissioned by the UK Department for International Development; 2012. As cited in, Paina, L., Wilkinson, A., Tetui, M. et al. (2017). Using Theories of Change to inform implementation of health systems research and innovation: experiences of Future Health Systems consortium partners in Bangladesh, India and Uganda. Health Research Policy and Systems, 15 (suppl 2), 109. https://doi.org/10.1186/s12961-017-0272-y

any phenomena disaggregated across different socio-economic sections, especially the vulnerable, and the differential impacts of the processes of change on them.

C. Methodological approaches

- **Analytical approaches**
    - **Historical analysis -** This would include tracing the processes of knowledge and institutional development and practice in health systems and their transformations. It is the study of past events, processes, and systems as emergent, non-linear, and interconnected phenomena as well as phase transitions that evolve through dynamic interactions among multiple agents, structures, and contingencies. It views history not as a linear sequence of cause-and-effect but as a complex adaptive system where small changes can lead to disproportionate outcomes, feedback loops shape trajectories, and path dependence influences future possibilities. This approach emphasizes the interplay of diverse factors—social, economic, cultural, technological and environmental—within specific contexts, acknowledging unpredictability, self-organization, and the co-evolution of systems over time.
    - **Political Economy of Health -** The "political economy of health" is concerned with how political and economic domains interact and shape individual and population health outcomes.[61]
    - **Analysis of social stratification, power & hierarchy** - Analysis of social inequalities, their determinants and their impact on health and health care.
    - **Epidemiological orientation** - Consideration of epidemiological context of the health system being studied, i.e. giving consideration to the nature and extent of health problems, their inter-relationship and determinants and thereby what the health system should address. The epidemiological context can also shape the formal and informal responses to health issues and therefore these interlinkages impact health systems.
    - **Knowledge pluralism -** The existence of different forms of knowledge related to individual and collective health, including the bio-medical and social sciences, the

[61] Harvey, M. (2021). The Political Economy of Health: Revisiting Its Marxian Origins to Address 21st-Century Health Inequalities, American Journal of Public Health, 111(2), 293-300. https://doi.org/10.2105/AJPH.2020.305996

several traditions of knowledge for understanding, improving and maintaining health such as Biomedicine, Ayurveda, Unani, Siddha, Traditional Chinese Medicine, Chiropractice, traditional birth attendants and home remedies.

  - **Medical pluralism -** Medical pluralism describes the availability of different medical approaches, treatments, and institutions that people can use while pursuing health: for example, combining biomedicine with so-called traditional medicine or alternative medicine.[62]
  - **Politics of Knowledge -** The privileging of certain forms of knowledge, and consequent de-legitimizing of other forms – under particular discourses and the resultant institutional arrangements is referred to as the Politics of Knowledge.[63] [64]

- **Operational Approaches**
  - **Interventional Study Design -** An investigation involving intentional change in some aspect of the status of the subjects, e.g., introduction of a preventive or therapeutic regimen or an intervention designed to test a hypothesized relationship.[65] In HSR, some of the common types of intervention study designs are[66]:
    - Experimental studies – Randomized Controlled Trials (RCT), Factorial Designs, Crossover Designs
    - Quasi-experimental studies – Non-randomized controlled studies, Interrupted time series, Regression discontinuity
    - Before –after (pretest - posttest) experimental studies – Single group pre-post design, Controlled before-after studies

---

62 Khalikova, V. (2021) 2023. "Medical pluralism". In The Open Encyclopedia of Anthropology, edited by Felix Stein. Facsimile of the first edition in The Cambridge Encyclopedia of Anthropology. Online: http://doi.org/10.29164/21medplural

63 Gaitonde R, et.al (2019) Some Thoughts on Health for All: the rationale for engaging with the politics of knowledge. MFC Bulletin, 380:9–15, https://www.mfcindia.org/mfcpdfs/MFC380.pdf

64 Priya R. (2023) "Co-producing and Pluralizing Health Knowledge for Re-visiting Development", In Dreams of a Healthy India: Democratic Health Care in Post-Covid Times, (eds.) Priya Ritu and Hameed Syeda, Vintage Penguin Random House, India, 2023:82-102.

65 Dictionary of Epidemiology, 5th Edition https://www.academia.dk/BiologiskAntropologi/Epidemiologi/PDF/Dictionary_of_Epidemiology__5th_Ed.pdf

66 Ram Bhandar, T. Health System Research: Development, Designs and Methods. Journal of Health and Allied Sciences, 2019;3(1), 68–72. https://doi.org/10.37107/jhas.60

- **Non-Interventional Study Design -** A study that does not involve any intervention (experimental or otherwise) on the part of the investigator.[67] In HSR, some of the common types of non- interventional study designs are[68 69]:
    - Exploratory studies: piloting studies, small scale comparative studies.
    - Descriptive studies: case studies, survey
    - Comparative/ analytical studies: cross-sectional comparative studies, case-control studies, cohort studies, comparing case studies.
    - Evaluation and System review: Cost analysis, document analysis, content analysis, Input-output-outcome analysis, Realist evaluation
    - Synthesizing Research - Meta-analysis, Systematic review, Narrative review, Realist review, Rapid reviews, Meta-ethnography review.

---

[67] Dictionary of Epidemiology, 5th Edition https://www.academia.dk/BiologiskAntropologi/Epidemiologi/PDF/Dictionary_of_Epidemiology__5th_Ed.pdf
[68] Ram Bhandar, T. Health System Research: Development, Designs and Methods. Journal of Health and Allied Sciences, 2019;3(1), 68–72. https://doi.org/10.37107/jhas.60
[69] Langlois, E.V., Daniels, K., Akl, E.A, editors. Evidence synthesis for health policy and systems: a methods guide. Geneva: World Health Organization; 2018. https://apps.who.int/iris/bitstream/handle/10665/275367/9789241514552-eng.pdf
