# Supplementary material for: The Dialogic Health Systems Research Framework (DHSRF): A tool for facilitating self-criticality, researcher interactions and knowledge management in Health Systems Research & Policy Studies
Source: PLOS Glob Public Health. 2025 Sep 19;5(9):e0004209. doi: 10.1371/journal.pgph.0004209 (PMC12449027; doi:10.1371/journal.pgph.0004209)
Supplement: Attachment — Submitted filename: Response to Reviewers_PLOS Global Public Health 050725.pdf [file pgph.0004209.s007.pdf]

Response to Reviewers

The line numbers mentioned below are from the Manuscript and the Supplementary Material 3: Glossary. For the line numbers mentioned for the manuscript, please refer to the clean copy.

**Reviewer No.**	**Reviewer Comment(s)**	**Authors' Response**	**Line numbers**
1	As a person, or perhaps the training I come from, I found the tools long and too structured- with too many rows and columns.	The tool's comprehensive design incorporates a wide range of options to serve as a flexible reference for reviewers and researchers. As noted in the paper (lines 398–404, 419-426; 585-591), not all components need to be addressed; reviewers should assess only those relevant to the study under consideration. This flexibility allows the tool to be tailored to the specific study and the reviewer's perspective.<br><br>The tool encourages introspection by making underlying values and conceptual foundations in HSR explicit, fostering dialogue to develop solutions fit for purpose and context. It includes most components found in HSR literature while remaining open-ended, enabling users to add or omit elements as needed.	Manuscript line nos. 398-404; 419-426; 585-591
1	I agree that there is merit in thinking about values in HSR, situating it in a research paradigm etc. But not always. This kind of thinking may suit core academic research (eg Phd work). But not always when eminent national research bodies (eg NHSRC) have to	While conceptualization and methodology are central to HSR, values and principles are often overlooked, despite their critical role in shaping research scope. For example, an HSR study prioritizing technical efficiency when evaluating a health systems intervention may not address equity. Misalignment between stated objectives and actual	

	produce HSR quickly. HSR also needs to quickly respond to situations, draw from tacit knowledge, and be rooted in practice. Those elements are missing from the framework as of now.	outcomes is common in HSR. A clear reference to values helps researchers clarify their intentions and ensure the research aligns with its goals. Rather than sidelining values for expediency, embracing value-critical reflection fosters more appropriate and sustainable solutions.<br><br>The Health Systems Research (HSR) framework integrates values, concepts, and methodologies essential for robust research. Incorporating these components into the Dialogic Health Systems Research Framework (DHSRF) can enhance the design of rapid studies, such as implementation research, by providing a ready reference for reflection. At the study design stage, the framework supports a conscious process of including and excluding various elements, enabling researchers to refine their design, acknowledge the likely strengths and limitations of the evidence that their choices will generate. Thereby, it should also strengthen the interpretation of findings by aligning them with the evidence.<br><br>The framework also acknowledges the role of tacit knowledge and practical experience, often rooted in conventional public health or pragmatist paradigms. It does not dismiss these but encourages dialogue between tacit knowledge and framework components during HSR design. Methodologically, capturing tacit knowledge about any of the elements included to answer the research questions is a common approach in HSR, and this framework does not exclude it.	

		With increased use, researchers will become more adept at applying the framework's components, enabling faster and more effective HSR execution. These components are familiar to trained health systems researchers and require only adaptation through practice. As learning health systems gain prominence, this framework supports mindful research and impactful solutions.	
1	Also, the complexities of systems thinking are not really present in the framework (only mentioned in the beginning). This could be a limitation.	While we believe the complexity components are incorporated within the framework as described below (for instance, lines 458-472 in the manuscript), we agree with the reviewer that these attributes need to be clearly articulated in the context of the framework. Hence, to ensure clarity, we have updated the glossary definitions for these components in the tool.<br><br>The tool integrates key attributes of a Complex Adaptive Systems (CAS) approach to enhance its applicability to Health Systems (HS) analysis. For example, path dependency is captured through historical analysis, reflecting how past events shape current system trajectories. Feedback loops and scale-free networks, observed in both formal (e.g., medical and professional associations) and informal (e.g., community healers' networks) HS segments, are addressed under the analysis of structures and processes for these elements. Emergent behaviors, whether in formal or informal settings, are similarly evaluated within these structures and	Supplementary material 3: Glossary line nos- 461-471; 330-336; 450-452

		processes. Phase transitions can be assessed through historical analysis and long-term theories of change. The modified glossary will help clarify these when using the tool.	
1	- It would be nice to incorporate the idea of beginning research from a 'real problem' in the field, and seeing if systems thinking and HSR in general as a way to help solve the problem.	We agree with the point being made. The paper does not begin with identifying a research problem, but rather the next step of developing a research design or reviewing research based on an already identified problem. However, we think this is a critical issue that needs exclusive attention, and we hope to take it up in a future publication.	
1	In the present form, the framework appears to be overly focussed on situating the research into a set of research paradigms, system elements, and values- which I feel may not always be necessary or relevant to do.	Public health encompasses diverse perspectives that shape how we prioritize problems, understand causality, propose solutions, and implement them. Hence dialogue across different perspectives, we feel, is essential.<br><br>The Covid-19 pandemic exposed critical flaws in the prevailing Health Systems Research. The dominant global health informed mechanistic 'one-size-fits-all approach', evident in the decision of nationwide lockdowns and the biomedical strategies focussed on narrowly defined risk factors, has been criticized for overlooking the social dimensions of public health. This oversight exacerbated social stressors, particularly for vulnerable populations, as highlighted extensively in literature. In this context, introspection about HSR practices is essential for course correction.	

		While policymakers and implementers bear some responsibility, researchers must also scrutinize their own contributions. The tool seeks to demystify HSR by providing an accessible template that unpacks its core components—research paradigms, system elements, and values. Such transparency would facilitatedialogue among researchers from varied trainings and schools of thought.<br><br>Although understanding HSR's theoretical foundations may involve a learning curve, familiarity with these concepts will enable more thoughtful research and contextually relevant solutions. Greater clarity in HSR and opportunities for peer dialogue are indispensable for advancing the field.	
2	The clarity on the concept of health systems is lacking in the paper as there are instances in the paper where HSR and HPSR are interchangeably used (p.5) while referring to AHPSR, which emerged in a different context. Whether HSR and HPSR are seen as similar by the authors need to be stated explicitly for better clarity. Those details in the supplementary material (S 2) about health systems need to be necessarily brought into the main paper for better clarity by reducing	The introduction section has been significantly modified as suggested for better clarity. Apart from rearrangements and addition of subheads, the following sections were added (Lines 92-112; 126-134; 146-148, 162-164)	Manuscript line nos 92-112; 126-134; 146-148; 162-164

	the introduction section.		
2	Second aspect is about the framework, whether proposing a uniform research framework for studying an event/ phenomenon, here the health systems, can also constrains the possibilities of research. By proposing a single research framework, here DHSRF, for studying an event/ phenomenon, it might bring in more rigidity in the idea of research itself. Rather for every research, it is important to make its epistemological and ontological assumptions explicit not only for ensuring rigour and validity of the methodology but also to understand the standpoint and purpose of the researcher. Unfortunately, that is missing in several of the current research and is true with the field of HSR as well. The three step tools of analysis mentioned in the framework resonates with the epistemological foundations that guide any research, namely, values and principles underlying the concepts of health systems and the actual conceptualisation, which is about the	We are not proposing any uniform research framework. What we, however, are attempting to do is to create a template for a common language that can foster dialogue between health systems researchers coming from varied perspectives: A dialogue around explicitly stated, "epistemological and ontological assumptions" of HSR informed by different "standpoint and the purpose of the researcher". As in the process of developing the tool, an iterative approach is paramount in its use too. As highlighted in the paper (lines 398-404; 419-426), not all components of the tool need to be addressed; reviewers should assess only those relevant to the study under consideration. This flexibility allows the tool to be tailored to the specific study and the reviewer's perspective. We believe the more the tool is used, the more it will get refined (Line 284-285).<br><br>We concur with the observations on the importance of surfacing the ontological and epistemological assumptions. In the tool, the values and principles, together with the methodological approach, represent the epistemological assessment (ways of knowing) of the ontological attributes (the way it is) of health systems i.e. Step two: the tool for analysing health systems conceptualisation. The paradigms, as informed by literature, involve both ontology and epistemology. These are clarified in the glossary	Manuscript line nos: 398-404; 419-426; 284-285<br><br>Supplementary material 3: Glossary-line nos: 110-128

	ontological assumptions and finally the methodology is about the paradigm. In the values and principles, it is the primary health care approach and the eco-social frameworks that is proposed, which is acceptable from a public health perspective. These three step tools can be organised better by separating ontological aspects of health systems from that of theoretical and methodological approaches and so on.		
2	Finally, every research does have diverse standpoints in its approach to the event/ phenomenon. Hence, the disciplinary standpoint for health systems research could be better justified if the public health disciplinary standpoint is adopted as central to health systems research. Health systems strengthening, people centredness, equity and sustainability then become characteristics of a public health disciplinary standpoint and can be proposed as the major purpose of all kinds of Health systems research. HSR historically was developed keeping the public health value	The public health disciplinary standpoint has been clarified in the text (Lines 230-238).<br><br>Some material from the supplementary file has been added to the manuscript (Lines 92-112; 126-134). However, the paper attempts to primarily introduce the framework (DHSRF) and open a dialogue around it among peers. Hence, it is focused on the framework mostly. Additional material that can help the reader delve deeper has been added to the supplementary materials and referred to in the main manuscript throughout.	Manuscript line nos: 230-238<br><br>Manuscript line nos: 92-112; 126-134

	system as central. It is the neoliberal context of individualism that has led to the domination of market values of economics and techno-managerial, and reductionism as the focus of HSR at the cost of public health standpoint. There is a need to regain it and can be demonstrated with the analysis of the existing studies on HSR as attempted in the analysis of the research papers in this article. Several of the content in the supplementary files need to be included in the paper for better clarity.		
3	Methodology section – It would be great if the authors indicated the overarching methodological approach for this process presented in this paper.	We cannot define a single overarching methodological approach for this study.<br>This study presents the Dialogic Health Systems Research Framework (DHSRF), developed using a multi-methods approach. The research tool development used a theory-based, iterative and reflexive methodology involving an initial formative systematic narrative review and a national expert consultation for feedback and validation.	Manuscript line nos: 230-238
3	In lines 236-241, the authors indicate that they found out that ‘similar research objectives and questions, the adoption of different paradigms lead to different	The above statement has been removed, and the content has been reworded for better conceptual clarity.	Manuscript line nos: 300-305

	conceptualisation of the problem…' this to me sounds rather upside down because usually conceptualisation of the problem comes before formulating research questions and questions and research objectives. I still believe the statement from the AHPSR methodology reader makes sense. I would consider rewording the above statement or removing it as it is rather confusing.		
3	The concept of ecosystem is not clearly articulated like other concepts. It would be useful for the researchers to shed more light on this.	The concept of ecosystem has been revisited, and more clarification has been added in lines (444-446) in the manuscript and in the Glossary in Supplementary File 3 (421-425)	Manuscript - Line nos: 444-446<br><br>Supplementary material 3: Glossary - line nos: 421-425
3	There is a small typo on line 536. Instead on 'This' , its currently 'his'.	This typo is corrected.	Manuscript - Line no: 615
