# Supplementary material for: The Dialogic Health Systems Research Framework (DHSRF): A tool for facilitating self-criticality, researcher interactions and knowledge management in Health Systems Research & Policy Studies
Source: PLOS Glob Public Health. 2025 Sep 19;5(9):e0004209. doi: 10.1371/journal.pgph.0004209 (PMC12449027; doi:10.1371/journal.pgph.0004209)
Supplement: Attachment — Submitted filename: Reviewer comments.docx [file pgph.0004209.s006.docx]

**Reviewer comments**

The authors have done a great and commendable work in creating this framework that consolidates the field of Health Systems Research (HSR). This framework will help to fill the gap in the HSR and bring researchers closer to speaking a more unified language. The interdisciplinary nature of this field is both an advantage and disadvantage. I therefore truly thank the researchers for the effort they put into this work and coming up with this comprehensive framework based on HRS work in India. The paper is well written and interesting to read. Well done!

Here are a few comments for the authors’ consideration.

1. Methodology section – It would be great if the authors indicated the overarching methodological approach for this process presented in this paper.
2. In lines 236-241, the authors indicate that they found out that ‘similar research objectives and questions, the adoption of different paradigms lead to different conceptualisation of the problem…’ this to me sound rather upside down because usually conceptualisation of the problem comes before formulating research questions and questions and research objectives. I still believe the statement from the AHPSR methodology reader makes sense. I would consider rewording the above statement or removing it as it is rather confusing.
3. The concept of ecosystem is not clearly articulated like other concepts. It would be useful for the researchers to shed more light on this.
4. There is a small typo on line 536. Instead on ‘This’ , its currently ‘his’.
